# Particulate matter 10 µm (PM_10_), 2.5 µm (PM_2.5_) datasets gathered by direct measurement, low-cost sensor and by public air quality stations in Fontibón, Bogotá D.C., Colombia

**DOI:** 10.1016/j.dib.2023.109323

**Published:** 2023-06-20

**Authors:** Karen Yulied Alfonso Albarracín, Astrid Altamar Consuegra, Jaime Aguilar-Arias

**Affiliations:** a Graduate Engineering Institute. Universidad Libre, Sede Bosque. Bogotá D.C. 111071, Colombia.; b Environmental Engineering Program. Universidad Libre, Sede Bosque. Bogotá D.C. 111071, Colombia.; c Chemical and Environmental Engineering Department, Universidad Nacional de Colombia. Sede Bogotá D.C. 111321, Colombia.

**Keywords:** Particle pollution, Data mining, Data science, Machine learning, Meteorological variables

## Abstract

Concentration of particulate matter directly affects air quality and human health. Three sources of information were used in this work to generate datasets on this matter at the Fontibón county in Bogota D.C., Colombia. The first source was a Davis AirLink^Ⓡ^ low-cost sensor air quality readings for PM_2.5_, PM_10_ and meteorological variables. The sensor was installed in the referred area, collecting air quality readings for PM_2.5_, PM_10_, as well as temperature, relative humidity, dew point, wet bulb, and heat index as meteorological variables during the months of May to August 2022. The second source was collecting by direct measurement the PM_10_ particles using a Tisch^Ⓡ^ Hi- Vol equipment, evaluated the concentration of particulate matter PM_10_ in the same place for 27 days. Finally, raw data was provided by the Bogotá’s Environmental District Bureau (SDA), validating in this work the data readings for the years 2021 and 2022 from the two meteorological stations located in the same county, named “*Fontibón*” and “*Móvil Fontibón*”, including Air quality data for PM_2.5_, PM_10_, Carbon Monoxide (CO), Ozone, Nitrogen Dioxide (NO_2_), Sulfur Dioxide (SO_2_) and the meteorological variables wind speed, wind direction, temperature, precipitation, relative humidity (RH) and Barometric pressure.

A Machine Learning model was made to perform the mining and completeness of the missing data with an iterative imputation and with a regression model, and the Pearson, Spearman and Kendall correlation coefficients were calculated, using Python language.

**Specifications Table**

**Table:** 

Subject	Data Science, Environmental Science, and Air Pollution
Specific subject area	Air quality, meteorological variables, data analytics.
Type of data	Figure Table
How the data were acquired	• Low-cost sensor data was acquired using a Davis AirLink^Ⓡ^ sensor for measurements of air quality and meteorological variables. • Direct measurement data was collected using a Tisch^Ⓡ^ TE-6070V Hi Vol equipment for PM_10_ air pollutant. • Public raw data was provided by the Bogotá’s Environmental District Bureau (SDA) for air quality and meteorological variables measurements of the stations “*Fontibón”* and “*Móvil Fontibón”*. The data was collected for the Fontibón County of Bogotá D.C., Colombia
Data format	Raw Analyzed
Description of data collection	Tisch^Ⓡ^ equipment and Davis AirLink^Ⓡ^ sensor were placed in accordance to the EPA specifications in an outdoor clear place, coordinates: (N 4°39’13.7”, W 74°6’50.7”), elevation: 2552 MASL. A dedicated wireless network was installed at the place using mobile internet service for connectivity of the Wi-Fi capable sensor. • Manual measurements were taken daily in the period from July 23 to August 18, 2022, (Filename: Raw_Consolidated_Manual_PM10.csv). • Sensor data readings were taken every 15 minutes in the period between May and August 2022 (Filename: Raw_Sensor_Consolidated.csv). • Data from air quality stations reported hourly in the period from January 01, 2021, to December 31, 2022 (Filename: Raw_Consolidated_ECA_Complete_2022.csv). • Raw data was filtered as follows: ○ For the sensor, were taken PM 2.5, (µg/m^3^), PM 10 (µg/m^3^), temperature (°F), relative humidity (%), dew point (°F), wet bulb (°F) and the heat index (°F). ○ For the air quality stations, the following columns were taken PM 2.5, (µg/m^3^), PM 10 (µg/m^3^), wind speed (m/s), wind direction (Degrees), temperature (°C), precipitation (mm) and relative humidity (RH %). • Description of the fields in the comma separated values (.csv) files is found in file named “*CSV files description.txt*”. • Data completeness was performed through an iterative imputation method.
	• Pearson, Spearman, and Kendall correlation coefficients were calculated. ○ *Consolidated_Air_Quality_Analysis_2021-2022_Manual.ipyb*: Google Collaboratory notebook with raw data filters, data completeness by iterative imputation, and Pearson, Spearman, and Kendall correlation coefficient calculations.
Data source location	• Institution: Universidad Libre - Sede Bosque Popular • City: Bogota D.C. • Country: Colombia • Raw data sources: ○ Dataset generated by the sensor. Location: N 4°39′13.7′′, W 74°6′50.7″. ○ Dataset gathered by direct measurement. Location: N 4°39′13.7′′, W 74°6′50.7". • Secondary Data sources: ○ Dataset provided by the SDA. Locations*:* *Fontibón* station: N 4°40′41.67′′, W 74°8′37.75″. *Móvil Fontibón* station: N 4°40′04.8′′, W 74°8′54.6″.
Data accessibility	Repository name: figshare Data identification number: 6439400 Altamar, Astrid (2023): Data_PM25. figshare. Collection. https://doi.org/10.6084/m9.figshare.c.6439400.v7 Direct URL to the data: https://figshare.com/collections/Data_PM25/6439400

## Value of the Data

• The data collection consists of measurements of air pollution by means of concentration of particulate matter PM_10_, PM_2.5_, and other pollutants, as well as readings of different meteorological variables, gathered from the Tisch^Ⓡ^ Manual equipment, Davis AirLink^Ⓡ^ sensor and from the public air quality stations in the Fontibón County in Bogotá, Colombia, in the time span from years 2021-2022. [1,2] These datasets are valuable source of information for the population in the surroundings, since air quality is a topic of crucial interest, considering the impact on health generated by breathing polluted air. Likewise, the data collected is useful for establishing correlations between the concentration of particulate matter and meteorological variables. [3]
• The inhabitants of sectors having harmful air quality index may consider data and methodology presented, in particular for the use of a low-cost option for the measurement of the concentration of particulate matter, for decision making and planning improvement actions in these locations to mitigate the possible risks that may be generated to health. The Datasets also provide information on the performance of low-cost sensors for registering information under different environmental conditions, and so, are a viable option to ensure the permanent measurement of air quality in places that require constant intervention due to their particulate matter concentration records. [4]
• Collected data may also be a source to analyze human activity impact on air quality, contrasting data for holidays, labor days and even the days during the Covid 19 pandemic shutdown.

## 1 Objective

Relevant information of the air quality is provided for a densely populated area with the lack of this data, collecting it from three different sources: direct measurement, low-cost sensors and public air quality stations in the Fontibón County in Bogota D.C., Colombia. Data analytics is applied to verify data integrity and to be a source for determining possible correlations between meteorological variables and air quality as PM_2.5_, PM_10_, concentrations.

## 2 Data Description

The datasets described in this section correspond to the treatment given to the readings gathered from: a) The Davis AirLink^Ⓡ^ sensor, collected from May to August 2022 (*Raw_Sensor_Consolidated.csv*), b) Data collected directly with the Tisch^Ⓡ^ TE-6070V Hi Vol equipment from July 23 to August 18, 2022 (*Raw_Consolidated_Manual_PM10.csv*) and c) Data collected by the air stations and provided by the SDA from January 01, 2021 to December 31, 2022 (*Raw_Consolidated_ECA_Complete_2022.csv*). All the equipment used for data collection was located in the Fontibón County in Bogota D.C.

### 2.1 Data Source 1: Davis AirLink^Ⓡ^ 7210 sensor

The Davis AirLink^Ⓡ^ sensor was factory calibrated S.N. 001D0A101387, Firmware Version Oct 21, 2020. The sensor has PM resolution: 1 mg/m^3^, accuracy: ±1 mg/m^3^. RH resolution: 0.1%, accuracy: ±2%.

The sensor has also been calibrated in accordance with the procedure indicated in Lewis et al. [5] by comparing measurements of the sensor to that of an official instrument located no more than 10 meters away, provided by the SDA.

Table 1 shows an excerpt of the raw data from the dataset for the Davis AirLink sensor on May 26, 2022, information is shown for all variables that were measured by the sensor, readings were recorded every 15 minutes in the period May-August 2022, the data is available in the file named *Raw_Sensor_Consolidated.csv*.

**Table 1:** Sample data from Davis AirLink^Ⓡ^ sensor data from May 26, 2022.

**Source:** Author. Raw_Sensor_Consolidated.csv.

The air quality variables included correspond to PM_2.5_ (µg/m^3^) and PM_10_ (µg/m^3^) particulate matter concentration, and the selected meteorological variables useful for their correlation. Table 2 shows the statistical analysis of these variables processed with data mining, since the raw data don't allow for the direct analysis, nor the calculation of correlation coefficients such as Pearson, Kendall, or Spearman. The statistical analysis includes the counting of variables to identify the need for data completeness, the arithmetic mean to identify the average of each variable and thus confirm that the data is in an acceptable range according to each type of variable, the standard deviation to evaluate the dispersion of the variables with respect to their mean, and the interquartile range taking the minimum and maximum values, to analyze the dispersion of the data compared with extreme and central values.

**Table 2:** Statistical analysis of air quality data and meteorological variables measured by the sensor Davis AirLink^Ⓡ^.

	PM_2.5_, μg/m^3^	PM_10_, μg/m^3^	Temp,°F	Hum,%	Dew Point,°F	Wet Bulb,°F	Heat index,°F
count	7864	7864	7864	7864	7864	7864	7864
mean	14.6	16.8	61.1	68.4	50.0	53.6	60.4
std	13.0	15.0	5.9	13.5	2.6	2.5	5.6
min	0	0	50	26	38	46	50
25%	5	5	56	59	48	52	56
50%	11	12	60	70	50	53	59
75%	22	25	65	79	52	55	64
max	417	418	81	99	58	63	80

**Source:** Author. Raw_Sensor_Consolidated.csv and Consolidated_Air_Quality_Analysis_2021_2022_Manual.ipynb.

**Fig. 1:**
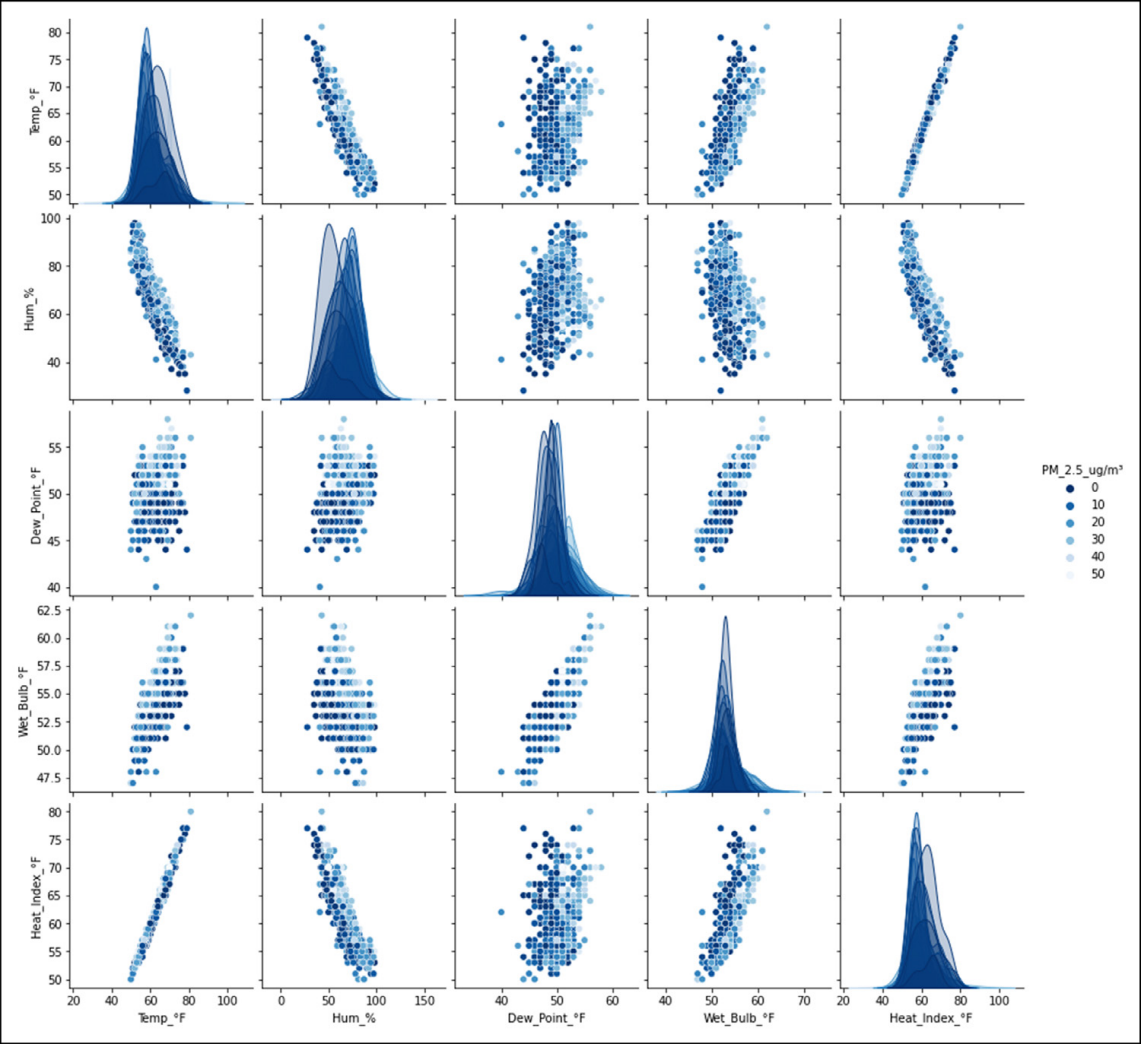
Relationship between the concentration of PM_2.5_ (µg/m^3^) and the meteorological variables measured by the Davis AirLink^Ⓡ^ sensor located in Fontibón County. **Source:** Author. Raw_Sensor_Consolidated.csv y Consolidated_Air_Quality_Analysis_2021_2022_Manual.ipynb.

Fig. 1 shows the relationship between the particulate matter PM_2.5_ (µg/m^3^) and the meteorological variables selected and processed with data mining; a matrix graph is used to visualize the dispersion of the relationship between the variables mentioned in a single graph.

**Fig. 2:**
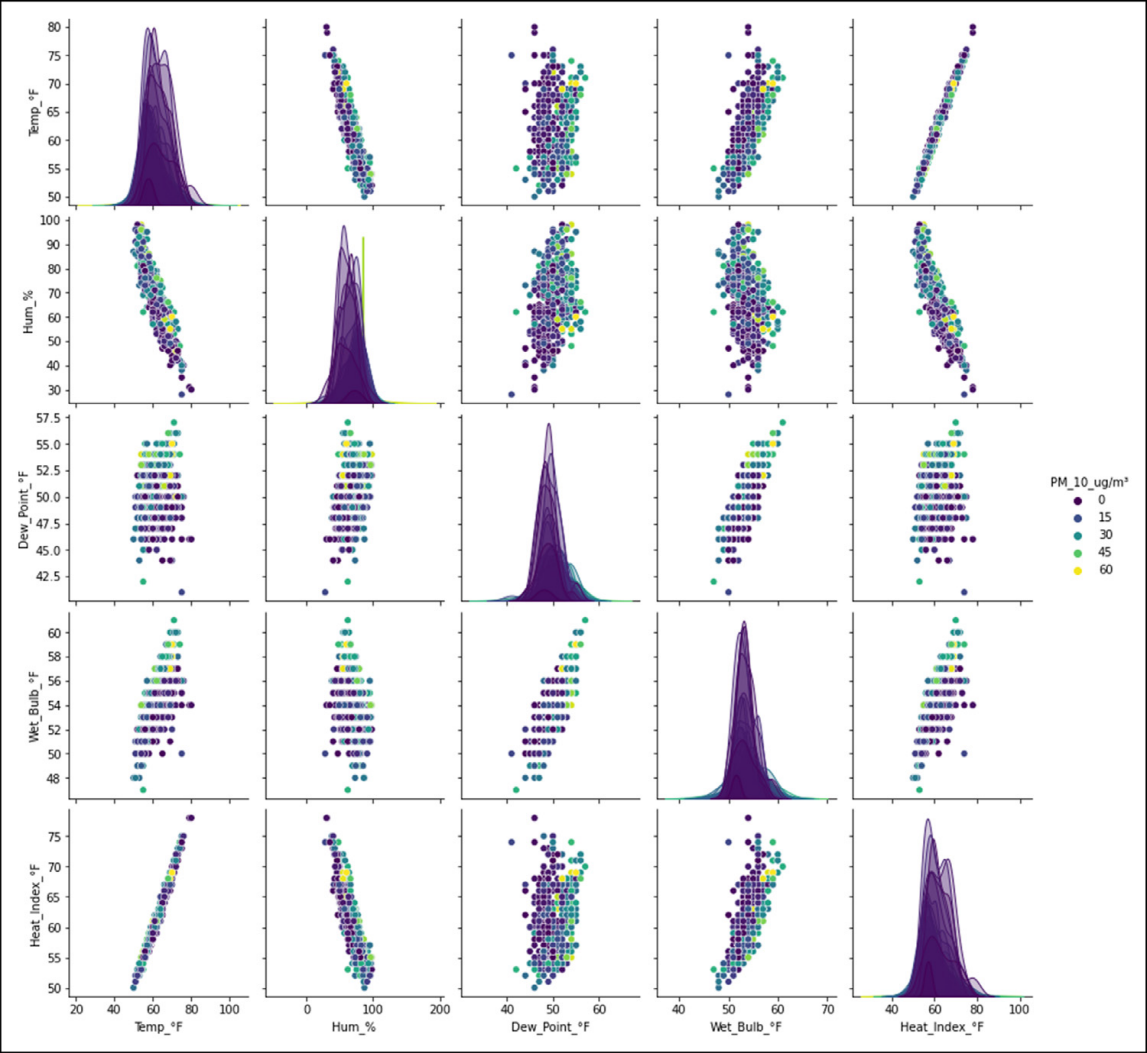
Relationship between the concentration of PM_10_ (µg/m^3^) and the meteorological variables measured by the Davis AirLink^Ⓡ^ sensor located in Fontibón County. **Source:** Author. Raw_Sensor_Consolidated.csv y *Consolidated_Air_Quality_Analysis_2021_2022_Manual.ipynb.*

Fig. 2 shows the relationship between the particulate matter variable PM_10_ (µg/m^3^) and the meteorological variables selected and processed with data mining, as in Fig. 1.

### 2.2 Data Source 2: Tisch^Ⓡ^ TE-6070V Hi Vol equipment

The Tisch^Ⓡ^ TE-6070V Hi Vol equipment was calibrated according to the maker's protocol, which relies on the certified orifice TE-5025, used to verify the flow rate (1.232±0.028 m^3^/min) passing through the quartz filter.

Table 3 shows an excerpt of the raw data for data collected by direct measurement from July 23 to 28, 2022, for the particulate matter PM_10_, data were collected daily at the same time of the day (11:00h), in the same location where the readings were taken with the Davis AirLink^Ⓡ^ sensor, the data is available in the file named *Raw_Consolidated_Manual_PM10.csv*.

**Table 3:** Sample data from PM_10_ (µg/m^3^) data collected directly over 27 days.

Date	Time, hh:mm	mf-mi, g	Pf, in_H_2_O_(Set_Up_reading)	Pf, in_H_2_O_(Pick_up_reading)	Pf_average, in_H_2_O	Pf, mmHg	Po/Pa	Ta,°C	Qa, m^3^/min	Qstd, m^3^/min	PM_10_, µg/m^3^
23/07/2023	11:00	0.0425	14.00	14.80	14.40	26.89	0.952	21.5	1.226	0.914	33
24/07/2023	11:00	0.0349	14.00	15.70	14.85	27.73	0.950	21.0	1.225	0.915	27
25/07/2023	11:00	0.0386	14.50	15.70	15.10	28.20	0.950	20.4	1.224	0.916	29
26/07/2023	11:00	0.0428	14.50	16.60	15.55	29.04	0.948	19.8	1.219	0.914	33
27/07/2023	11:00	0.0450	13.20	16.50	14.85	27.73	0.951	20.9	1.225	0.915	34
28/07/2023	11:00	0.0634	12.60	16.40	14.50	27.08	0.952	18.9	1.222	0.919	48

**Source:** Author. Raw_Consolidated_Manual_PM10.csv.

Table 4 shows the statistical analysis of the particulate matter PM_10_ concentration of air quality, and the selected meteorological variables with the same criteria as above. When performing a preliminary analysis of the data obtained based on data mining, it was observed that the data did not require additional processing, since they presented good data quality. [6].

**Table 4:** Statistical analysis of air quality data and variables measured by direct collection.

	PM_10_, μg/m^3^	Pf, mmHg	Po/Pa	Ta,°C
count	27	27	27	27
mean	33.1	28.09	0.950	20.49
std	11.5	0.54	0.001	1.88
min	19.7	26.89	0.948	17.40
25%	24.4	27.73	0.949	19.05
50%	30.6	28.11	0.950	20.40
75%	39.6	28.39	0.951	21.40
max	68.3	29.14	0.952	24.60

**Source:** Author. Raw_Consolidated_Manual_PM10.csv and Consolidated_Air_Quality_Analysis_2021_2022_Manual.ipynb.

**Fig. 3:**
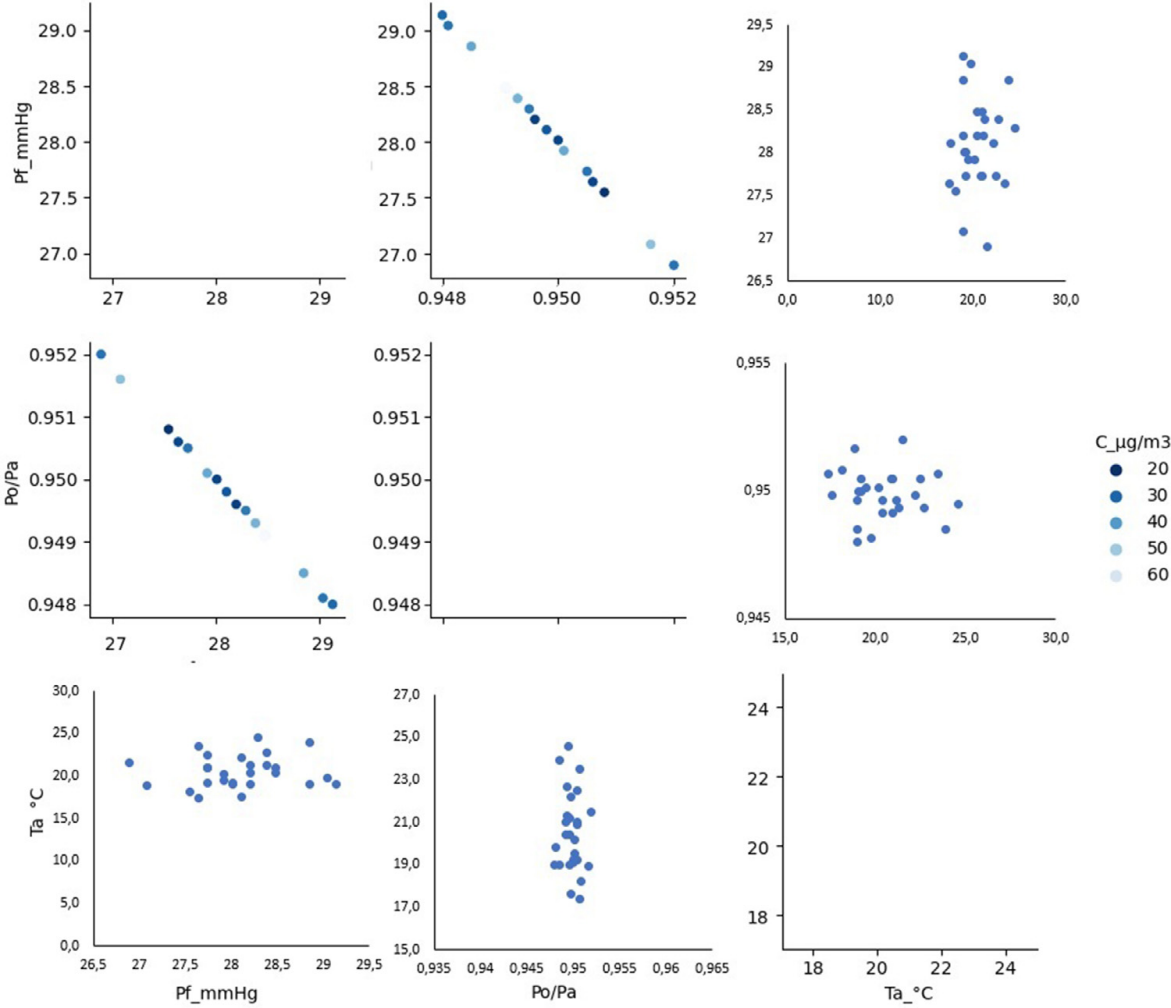
Relationship between the concentration of PM_10_ (µg/m^3^) and the variables measured by direct collection in Fontibón County. **Source:** Author. *Raw_Consolidated_Manual_PM10.csv* and *Consolidated_Air_Quality_Analysis_2021_2022_Manual.ipynb.*

Fig. 3 shows the relationship between the air quality variables and the selected meteorological variables for the Tisch^Ⓡ^ TE-6070V Hi Vol equipment. The high relevance of these data relies on the fact that they were obtained from the direct measurement of the particulate matter collected from the air with high confidence, in contrast to the indirect measurement method utilized by the electronic sensors, which constitutes a valuable contribution, not only for the information provided itself, but for providing data using a reference technique on air quality pollutants.

### 2.3 Data source 3: Bogota's Environmental District Bureau (SDA)

Raw data provided by the SDA in Bogotá-Colombia, contains air quality information measured hourly for the full years 2021 and 2022 stored in two different files. It should be noted that before using the data for its corresponding analysis, it is necessary to evaluate the quality of the data, due to occurrence of missing records.

The two raw data files provided for processing were the first for the meteorological variables and the other for the pollutants, so they were merged and consolidated from January 01/2021, to December 31/2022 in a single file called *Raw_Consolidated_ECA_Complete_2022.csv*. Table 5 shows an example of the raw data from the air quality stations. These air quality stations were located in the same County (Fontibón) as the other sources, allowing future analysis of data of air quality variables and meteorological variables.

**Table 5:** Sample data of the information from the air quality stations sent by the Bogotá’s Environmental District Bureau (SDA) years 2021–2022.

Date	Time, hh:mm	Station	PM_10_, µg/m^3^	CO, ppm	OZONE, ppb	NO_2_, ppb	SO_2_, ppb	PM_2.5_, µg/m^3^	Wind Speed, m/s	Wind Direct, Degrees	Temperature, °C	Precipitation, mm	HR, %	Baro Press, mmHg
1/01/2021	1:00	FONTIBÓN	40.3	0.491	8.943	16.291	0.858	36.8	1.2	322.0	13.3	NaN	86.29	NaN
1/01/2021	2:00	FONTIBÓN	92.5	0.446	7.808	16.837	0.985	79.5	2.7	358.0	13.3	NaN	85.06	NaN
1/01/2021	3:00	FONTIBÓN	68.3	0.511	5.678	17.262	1.106	62.5	1.7	355.0	13.4	NaN	83.89	NaN
1/01/2021	4:00	FONTIBÓN	61.3	0.498	5.873	15.462	1.021	54.6	1.8	337.0	13.2	NaN	84.09	NaN
1/01/2021	5:00	FONTIBÓN	63.9	0.484	6.249	14.567	0.909	53.1	2.0	331.0	13.1	NaN	85.14	NaN

**Source: SDA.** Raw_Consolidated_ECA_Complete_2022.csv.

Table 6 shows the statistical analysis of the data provided for the public air quality stations, showing the amount of data in the second row (being evident the requirement of data processing), the mean and standard deviation to visualize the dispersion of the data and the interquartile range for the measurement of the dispersion with respect to the minimum and maximum points. [6].

**Table 6:** Statistical analysis of the data from the air quality stations provided by the SDA.

	PM_2.5_, µg/m^3^	PM_10_, µg/m^3^	Wind Speed, m/s	Temperature_°C	Precipitation_mm	RH_%
count	30711	30711	23233	24797	12410	24784
mean	19.38	43.77	2.91	15.10	0.11	69.49
std	11.84	26.29	2.19	2.68	0.82	14.33
min	0.1	0.1	0	7.2	0	18
25%	10.2	24.9	1.2	13.1	0	59
50%	17.3	38.5	2.3	14.6	0	73
75%	26	57.3	3.9	17.1	0	81
max	115.3	277.1	12.9	23.9	32.7	94

**Source: SDA** Raw_Consolidated_ECA_Complete_2022.csv and Consolidated_Air_Quality_Analysis_2021_2022_Manual.ipynb.

**Fig. 4:**
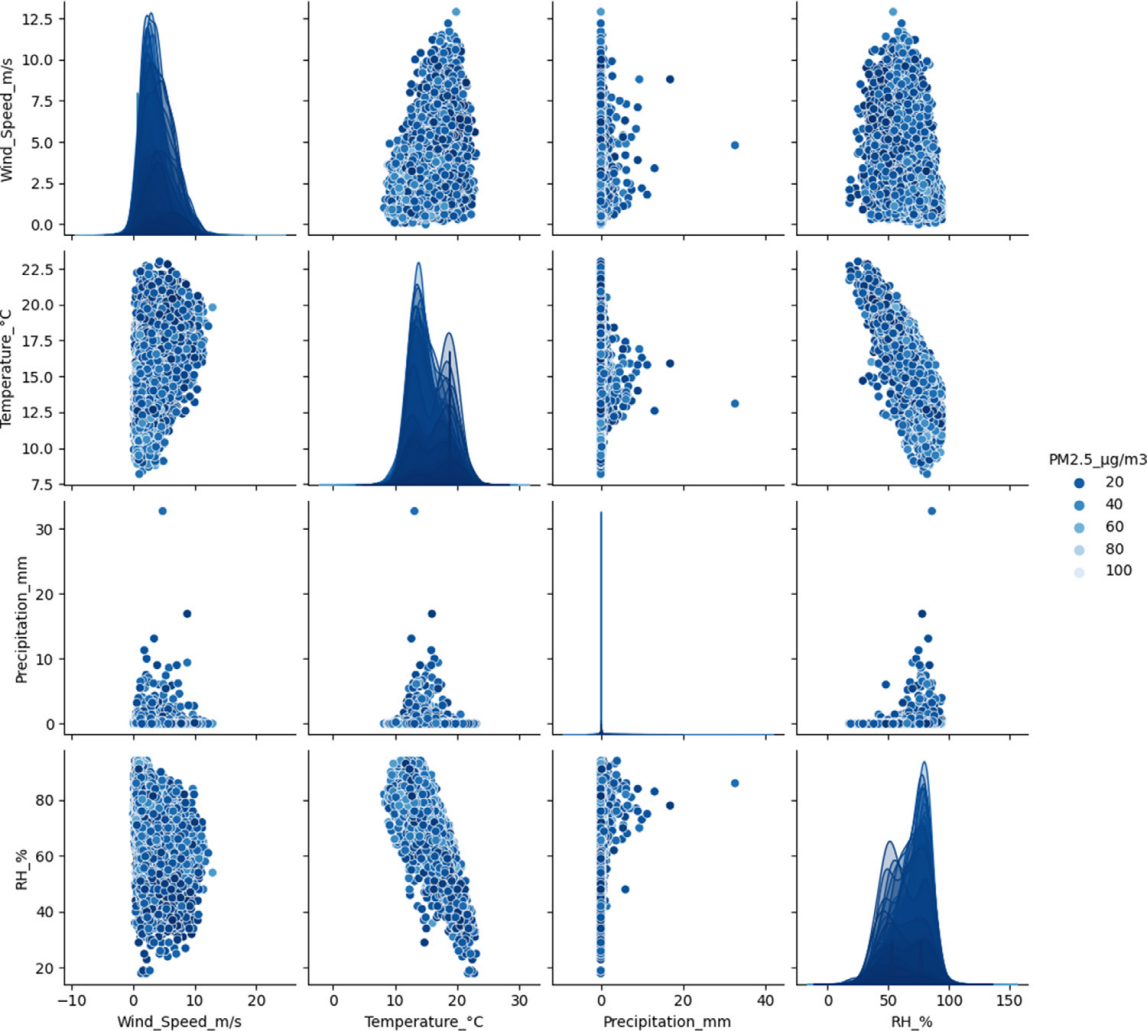
Relationship between the concentration of PM_2.5_ (µg/m^3^) and the meteorological variables measured by the air quality stations of the town of Fontibón.

**Source:** SDA. Author. Raw_Consolidated_ECA_Complete_2022.csv and Consolidated_Air_Quality_Analysis_2021_2022_Manual.ipynb.

**Fig. 5:**
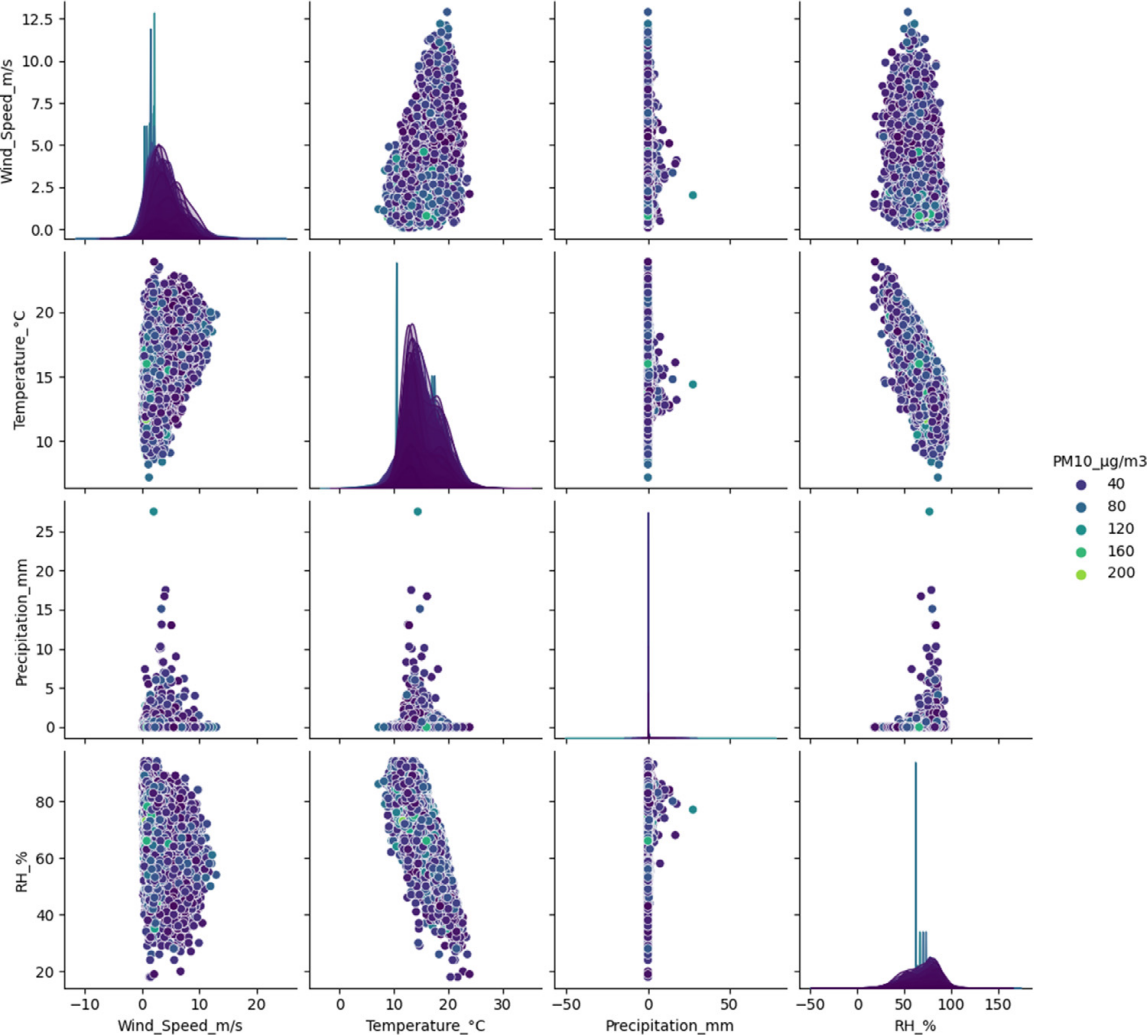
Relationship between the concentration of PM_10_ (µg/m^3^) and the meteorological variables measured by the air quality stations in Fontibón County. **Source: SDA. Author.***Raw_Consolidated_ECA_Complete_2022.csv* and *Consolidated_Air_Quality_Analysis_2021_2022_Manual.ipynb.*

Fig. 4, Fig. 5 show the relationship between the PM_2.5_ and PM_10_ particulate matter concentration respectively, and the selected meteorological variables, based on the data from the air quality stations processed with data mining and standardized to show in a clever way the dispersion of the data and a perspective of the relationship between each of the variables.

## 3 Experimental Design, Materials and Methods

### 3.1 Data Source: Tisch^Ⓡ^ TE-6070V Hi Vol equipment

The equipment used is classified as an Hi Vol active manual sampler, in this case the TISCH^Ⓡ^ TE-6070V Volumetric flow controlled (VFC) PM_10_, US EPA certified. Calibration protocol was performed using the Tisch^Ⓡ^ certified orifice TE-5025, verifying the flow rate passing through the quartz filter. The analytical balance used for weighing the filters before and after the sampling was also calibrated.

High volume air sampler model TE-6070V Hi Vol from Tisch^Ⓡ^ collects large quantities of particles ranging from 0.1 to 1 gram, mass of solid particles collected from the high flow rate for the sampling period of 24h, facilitating gravimetric analysis with confidence. The protocol followed for the direct measurements was according to the Reference Method for the Determination of Particulate Matter as PM_10_ in the Atmosphere [7].

The equipment location was chosen considering: the distance to the main road, distance to borders, height to the floor of the sampler location, and safe distance to the closer trees and buildings to avoid interferences [8]. In this case, the equipment was installed in a place located in an urban area meeting all these requirements: coordinates: N 4°39′13.7′′, W 74°6′50.7′′, elevation: 2552 MASL, height to the floor of the sampling chamber: 2.5 m. Average temperature: 12°C, Average barometric pressure: 564 mbar.

In the active high-volume sampler, a measured amount of ambient air was drawn into a sample box through a quartz filter for 24 hours. The filter is weighed before and after to determine the net weight change. The total volume of air sampled is determined from the average flow velocity and the sampling time. The total concentration of particles in ambient air is calculated as the mass collected divided by the volume of air sampled, adjusted to reference conditions. This process was repeated during 27 consecutive days in the period of 07/23/2022 – 08/18/2022.

The quartz filters were dried before sampling, placing them inside a chamber with abundantly dry silica gel for 2 weeks to absorb moisture, monitoring the filters weight on an analytical balance until constant weight readings, so that the filters were dry enough.

The daily recorded data from the air quality station, such as the initial and final weight of the filters, the readings from the hour meter of the equipment, as well as the initial and final stagnation pressure, wind speed, temperature, relative humidity, dew point, atmospheric pressure and wind direction, measurements were consolidated in an electronic worksheet to calculate the daily PM_10_ concentrations according to the calculation procedure established in the reference method.

### 3.2 Data Sources: Davis AirLink^Ⓡ^ sensor and Public stations SDA

The Davis AirLink^Ⓡ^ sensor was installed in the same place as the Tisch^Ⓡ^ equipment, coordinates: N 4°39′13.7′′, W 74°6′50.7′′, elevation: 2552 MASL, height to the floor of the sampling chamber: 2.5 m. Average temperature: 12°C, Average barometric pressure: 564 mbar. Data from Public stations belonging to the Air Quality Monitoring Network (RMCAB) managed by the SDA in Bogotá: *Fontibón* station is located N 4°40′41.67′′, W 74°8′37.75′′, elevation: 2551 MASL, height: 11 m. The *Móvil Fontibón* station is located N 4°40′04.8′′, W 74°8′54.6′′, elevation: 2547 MASL, height: 10 m.

Calibration of the low-cost sensors is currently under development by the European Committee for Standardization (CEN), in this work the methodology proposed by Lewis et al. [5] also named as ‘Collocation’ was followed, consisting of comparing measurements of the Davis AirLink^Ⓡ^ sensor to that of an official instrument located no more than 10 meters away, provided by the SDA.

Treatment for the data was necessary both for the gathered from the Davis AirLink^Ⓡ^ sensor, and for those provided by the SDA from the air quality stations [9]. This treatment is discussed hereafter.

The file *Consolidated_Air_Quality_Analysis_2021_2022_Manual.ipynb*, contains the Python code for the analysis. A description of the data from each of the sources, as well as the completeness of the missing data by means of imputation methods with the technique of Machine Learning called KNNImputer available in the Python's library *sklearn.impute*, which takes the nearest neighbor data to impute and define the missing value. Once the data was completed, the graph of the relationship between the air quality measured by the concentration of particulate matter PM 2.5 and the meteorological variables chosen for the study was made, both for the data from the air quality stations and for the data generated by the Davis AirLink^Ⓡ^ sensor. [10]

Likewise, the calculation of the Pearson, Spearman and Kendall correlation coefficient was performed for the concentration of particulate matter PM 2.5 in relation to each of the meteorological variables established in each data set.

Finally, a training was carried out on the models of linear regression, regression by nearest neighbors and regression with support vectors, comparing the results of these and taking as reference the data set of the air quality stations, considering that they had a greater number of records, which allowed their training and evaluation of the prediction.

## Ethics Statements

Raw Data for this work were partially obtained from the Bogotá’s Environmental District Bureau, as well as from the low-cost Davis AirLink^Ⓡ^ sensor owned by the authors.

This study does not involve research with animals or humans.

## CRediT authorship contribution statement

**Karen Yulied Alfonso Albarracín:** Formal analysis, Data curation, Writing – original draft, Visualization. **Astrid Altamar Consuegra:** Supervision, Conceptualization, Methodology, Writing – review & editing. **Jaime Aguilar-Arias:** Supervision, Writing – review & editing.

## Declaration of Competing Interests

The authors declare that they have no known competing financial interests or personal relationships that might have appeared to influence the work reported in this paper.

## Data Availability

Análisis_Calidad_aire_Consolidado_2021_2022_Manual.ipynb (Original data) (Figshare). CONSOLIDADO_ECA_COMPLETO_2022.csc (Original data) (Figshare). Consolidado_Sensor.csv (Original data) (Figshare). CONSOLIDADO_MANUAL_PM10.csv (Original data) (Figshare).

## References

[1] Badura M., Batog P., Drzeniecka-Osiadacz A., Modzel P. (2018). Evaluation of low-cost sensors for ambient pm2.5 monitoring. J. Sens..

[2] Castell N., Dauge F.R., Schneider P., Vogt M., Lerner U., Fishbain B., Broday d., Bartonova A. (2017). Can commercial low-cost sensor platforms contribute to air quality monitoring and exposure estimates?. Environ. Int..

[3] Dimakakou E., Johnston H., Streftaris G., Cherrie J. (2018). Exposure to environmental and occupational particulate air pollution as a potential contributor to neurodegeneration and diabetes: a systematic review of epidemiological research. Int. J. Environ. Res. Public Health.

[4] Kelly K.E., Whitaker J., Petty A., Widmer C., Dybwad A., Sleeth D., Martin R., Butterfield A. (2017). Ambient and laboratory evaluation of a low-cost particulate matter sensor. Environ. Pollut..

[5] Lewis A., Peltier W., von Schneidemesser E. (2018).

[6] Omokungbe O.R., Fawole O.G., Owoade O.K., Popoola O.A.M., Jones R.L., Olise F.S., Abiye O.E. (2020). Analysis of the variability of airborne particulate matter with prevailing meteorological conditions across a semi-urban environment using a network of low-cost air quality sensors. Heliyon.

[7] Electronic Code of Federal Regulations (eCFR). Reference Method for the Determination of Particulate Matter as PM10 in the Atmosphere. Title 40 - CHAPTER I - SUBCHAPTER C - PART 50 - Appendix J. URL: https://www.ecfr.gov/current/title-40/chapter-I/subchapter-C/part-50/appendix-Appendix J to Part 50. Updated: Feb. 23 2023; Accessed 26 February 2023.

[8] Tirado, D. Evaluación de la calidad del aire por material particulado PM 10 en una zona del barrio ciudad salitre en la localidad de Fontibón (UPZ 110). December, 2022. https://repository.unilibre.edu.co/handle/10901/23665.

[9] Sayahi T., Butterfield A., Kelly K.E (2019). Long-term field evaluation of the plantower PMS low-cost particulate matter sensors. Environ. Pollut..

[10] Chen L.-J., Ho Y.-H., Lee H.-C., Wu H.-C., Liu H.-M., Hsieh H.-H., Huang Y-T., Lung S.-C.C. (2017). An open framework for participatory PM2.5 monitoring in smart cities. IEEE Access.

